# Lessons, Challenges and Future Therapeutic Opportunities for PI3K Inhibition in CLL

**DOI:** 10.3390/cancers13061280

**Published:** 2021-03-13

**Authors:** Valerio Guarente, Paolo Sportoletti

**Affiliations:** Department of Medicine and Surgery, Institute of Hematology-Centro di Ricerca Emato-Oncologica (CREO), University of Perugia, 06129 Perugia, Italy; valerio.guarente@studenti.unipg.it

**Keywords:** PI3K inhibitors, chronic lymphocytic leukemia, targeted therapy

## Abstract

**Simple Summary:**

The phosphoinositide 3-kinase (PI3K) is a family of kinases that play a key role in the biology of chronic lymphocytic leukemia (CLL). Inhibitors of PI3K demonstrated efficacy in the treatment of CLL, associated with significant adverse events that limited the clinical use of this drugs. In this review, we underlined the relevance of PI3K inhibitors in CLL, we collected recent data about the use of these molecules in clinical practice and in clinical trial discussing strategies for the management of adverse events, which could help to improve the use of these therapies in the treatment of CLL.

**Abstract:**

Chronic lymphocytic leukemia (CLL) shows constitutive phosphatidylinositol 3-kinase (PI3K) activation resulting from aberrant regulation of the B-cell receptor (BCR) signaling. PI3K inhibitors have been evaluated in CLL therapy, bringing a new treatment opportunity for patients with this disease. Despite the proven therapeutic efficacy, the use of approved PI3K inhibitors is limited by severe immune-mediated toxicities and given the availability of other more tolerable agents. This article reviews the relevance of PI3K signaling and pharmacologic inhibition in CLL. Data on efficacy and toxicity of PI3K inhibitors are also presented, as well as strategies for overcoming barriers for their clinical use in CLL treatment.

## 1. Introduction

Chronic lymphocytic leukemia (CLL) is a slow-growing cancer that primarily afflicts elderly population and represents the most common type of leukemia in Western countries [1]. CLL induces B-cell expansion in blood, bone marrow and secondary lymphoid tissues that is primarily driven by B-cell receptor (BCR) signaling.

The phosphoinositide 3-kinase (PI3K) family plays a key node in BCR pathway promoting proliferation and survival in B cell malignancies, including CLL [2]. Advances in our understanding of the role of PI3K in CLL led to the development of modern pathway inhibitors which are revolutionizing the treatment landscape of this disease. The main targets for PI3K inhibition are the four isoforms α, β, γ, and δ, collectively regulating CLL cell chemotaxis, cytoskeletal rearrangement and the interaction of leukemic cells with the tumor microenvironment.

The potential efficacy of small molecule PI3K inhibitors (PI3Kis) has been widely recognized in CLL therapy [3]. However, several issues have become pertinent with the availability of PI3K inhibitors and the limits arising are of similar magnitude as the advances that have been made. In particular, toxic side effects can significantly impair patients’ quality of life as well as treatment efficacy.

In this manuscript, we will review the scientific basis for the use of PI3K inhibitors as treatment for CLL. We will describe efficacy and side effects of molecules currently employed in clinic or under development, providing strategies to overcome treatment challenges.

## 2. The PI3K in CLL: BCR-Signaling and Beyond

PI3Ks represent a family of highly conserved lipid kinases which generate second messengers by specific catalytic 3-hydroxy phosphorylation of phosphatidylinositol (PI) [4]. PI3Ks are divided into four classes based on their in vitro lipid substrate specificity and structure [5]. Class I is the subfamily most implicated in human cancer and functions as a dimeric enzyme consisting of catalytic and regulatory subunits. Mammals express four tissue-specific Class I catalytic isoforms (p110 α, β, γ and δ) that are associated with various tissue pathways. PI3K isoforms p110α and p110β are ubiquitously expressed [6,7,8], while γ and δ isoforms are highly enriched in leukocytes [9,10,11], where they have distinct and nonoverlapping roles in immune cell development and function. Therefore, tissue distribution informs the expected activity and toxicity seen with pharmacologic inhibition of these different isoforms.

**Figure 1:**
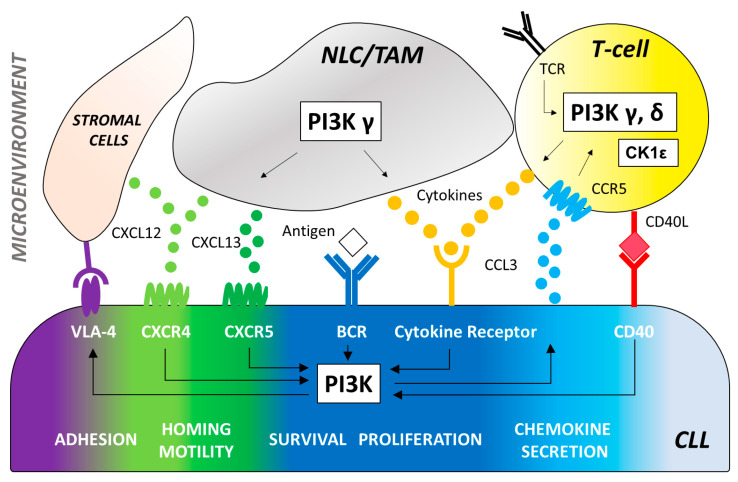
The effector molecule PI3K plays a central role in multiple signaling pathways promoting B-cell development and function. CLL cells depend on signaling via the BCR engagement that induces the activation of PI3K-dependent pathways to increase survival, proliferation, adhesion to stromal cells (via VLA-4), and secretion of chemokines (CCL3 and CCL4). Antigens for the BCR may be expressed by the CLL cells themselves or by other cells in the microenvironment. Key cell types of the tumor microenvironment include: 1. T-cells that can be recruited by CCL3 production and CCR5 receptor binding to provide CD40 ligand (CD40L) and cytokines (IL4, TNFα, IFNγ, CCL3, CCL4) that induce PI3K activation and stimulate the expansion of the malignant CLL clone. PI3K/AKT signaling triggered by the T cell receptor (TCR) plays a critical role in T-cell differentiation, function, and immune tolerance. PI3Kδ controls the activity of regulatory T-cells (Tregs) resulting in enhanced anti-tumoral immune functions which may contribute to the activity and toxicity of PI3Kδ inhibitors in CLL therapy. Impairment of Treg function by PI3Kδ inhibition can be counteracted with CK1ε modulation. 2. Myeloid-derived nurse-like cells (NLC) and tumor-associated macrophages (TAM) that secrete cytokines and chemokines, such as CXCL12 and CXCL13, which bind to CXCR4 and CXCR5 on CLL cells. PI3Kδ is a central integrator of signals from the CXCR4/CXCL12 and CXCR5/CXCL13 axis. 3. Stromal cells of mesenchymal origin that also secrete CXCL12 to facilitate CLL recruitment to and retention in the lymph nodes via VLA-4 binging. *VLA-4 = Very late antigen 4, CCL3 = C-C motif chemokine ligand 3, CCL4 = C-C motif chemokine ligand 4, CCR5 = C-C motif chemokine receptor type 5, CD40L = CD40 ligand, IL4 = Interleukin 4, TNFα = Tumor necrosis factor, INFγ = Interferon gamma, CXCL12 = C-X-C motif chemokine ligand 12, CXCL13 = C-X-C motif chemokine ligand 13, CXCR4 = C-X-C chemokine receptor type 4, CXCR5 = C-X-C chemokine receptor type 5.

PI3Ks are a part of the intracellular PI3K/AKT/mammalian target of the rapamycin (mTOR) signaling axis that plays a key role in signal transduction, cell metabolism and survival [8,12]. Importantly, PI3K lies downstream of the B-cell antigen receptor signaling in both normal and malignant B cells (Figure 1), the latter being described to have a constitutively activated PI3K/AKT/mTOR pathway [13].

CLL cells with unmutated immunoglobulin heavy chain variable region (IGHV) show significantly greater PI3K expression compared to IGHV mutated counterpart [14]. After BCR engagement, the Class I PI3Ks p110γ and p110δ produce the phospholipid phosphatidylinositol-(3,4,5)-trisphosphate (PIP3) [15,16] leading to the activation of several downstream mediators of essential cellular processes, including cell survival, proliferation, and metabolic fitness [11,17]. In B cells, the PH domain of both AKT and BTK allow PIP3 binding to keep an activation loop “open” for substrate docking [18]. Additional downstream effects of PI3K activation include inhibition of IκB Kinase (IKK), Forkhead box O (FOXO), and the proapoptotic protein B-cell lymphoma 2 (BCL2) associated agonist of cell death (BAD) as well as activation of mTOR. The PI3K/AKT signaling can also inhibit the lysosome-mediated degradation of NOTCH1 [19] a new key cancer gene in CLL whose genetic and pathway alterations are likely to represent a novel oncogenic process in this disease [20,21,22,23]. Interestingly, we demonstrated the involvement of the PI3Kδ oncogenic pathway in the phosphorylation of NOTCH1 intracellular domain in CLL [24,25,26]. Besides its role in BCR signaling, PI3Kδ also plays an important role in CLL cell migration and tissue homing. In vitro studies have shown that antigen receptors, costimulatory molecules, cytokine and chemokine receptors signaling can all trigger an increase in PIP_3_ and phosphorylation of AKT in different B-cell disease [27,28,29] (Figure 1).

The PI3K pathway extends beyond the direct regulation of CLL cell-intrinsic activities through the BCR (Figure 1). It is well known that the tumor biology of CLL cells depends on a complex cross talk with a number of non-neoplastic cells comprising the tumor microenvironment (i.e., mesenchymal stromal cells, nurse-like cells and lymphoma-associated macrophages, in concert with T cells, natural killer cells and extracellular matrix components). Several studies highlighted the relevance of specific PI3K isoform signaling on CLL microenvironment. The PI3K p110δ is critical for survival and function of CD4^+^CD25^+^FOXP3^+^ regulatory T cells (Treg) [30,31]. In mice, PI3Kδ deletion had a significant deleterious effect on the induction of Treg which cannot block the development of experimental colitis [16,32]. Thus, the role of p110δ Treg function may explain some of the toxicities seen with PI3K inhibitors describe thereafter in this review. Interestingly, immune suppression of Tregs can be restored by the inhibition the casein-kinase 1 epsilon (CK1ε), a Wingless-related integration site (WNT) signaling amplifier that negatively modulates regulatory T cell function [33]. This represents an alternative approach to lower the risk of immune-mediated toxicities frequently observed with PI3K p110δ inhibitors. PI3Kγ and δ are essential mediators of chemokine receptor signaling necessary for the interaction of CLL with tissue-resident stromal cells. Specifically, PI3Kγ contributes to the differentiation and migration of key support cells in the tumor microenvironment, such as CD4^+^ T cells and M2 tumor-associated macrophages, which sustain leukemia cells in a protective niche [34,35]. PI3K p110γ is also critical for many aspects of neutrophil function, including chemotaxis, phagosome formation, and the oxidative burst [36,37]. Additionally, genetic p110δ inhibition enhances toll-like receptor signaling in macrophages [32] and leads to prolonged pro-inflammatory responses in dendritic cells [38]. These data indicate that PI3Ks play a critical role in the innate immune systems as well as the adaptive immune system, suggesting that p110δ and p110γ inhibition may enhance immune responses. PI3K inhibitors efficacy and toxicity is the result of a combination of these pleiotropic effects in the tumor microenvironment with CLL cell-intrinsic activities.

## 3. Approved PI3K Inhibitors in CLL

### 3.1. Idelalisib

**Figure 2:**
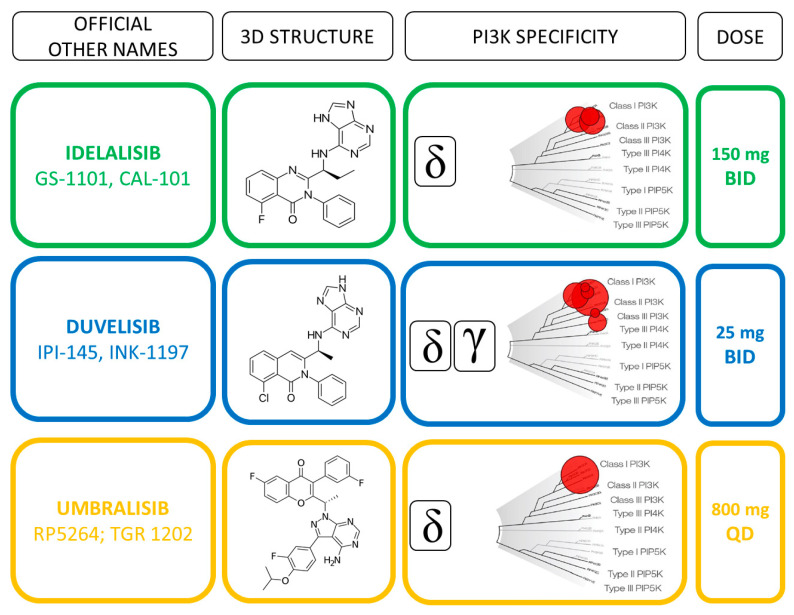
Direct comparison of idelalisib, duvelisib and umbralisib profiles.

Idelalsib (CAL-101) is a first-generation inhibitor of the delta isoform of the catalytic subunit of PI3K p110δ, which is preferentially expressed in leukocytes [39] (Figure 2). 

Idelalisib inhibited cellular pathways involved in the homing and retention of normal and malignant B cells in lymph nodes and bone marrow, thereby causing transient lymphocytosis and rapid lymph node shrinking [40,41]. The recommended dosage of idelalisib is 150 mg administered orally twice daily. A phase III study evaluated idelalisib and rituximab versus placebo and rituximab therapy in relapsed CLL, with an overall response rate (ORR) of 81% that allowed the first FDA and EMA approval of a PI3K inhibitor in CLL [3]. In a second trial, a population of treatment naïve (TN) elderly patients with *TP53* alterations displayed 100% ORR with high PFS and OS rates at 36 months [42]. A recent phase III randomized trial (ASCEND) assessed the efficacy and safety of acalabrutinib compared with idelalisib plus rituximab (idelalisib-R) for R/R CLL [43]. This trial, which is one of the first direct comparison between two inhibitors of the B-cell receptor pathways, demonstrated significantly longer PFS of acalabrutinib monotherapy over idelalisib-R regimen. Acalabrutinib was discontinued in 11% for AEs while idelalisib-R was discontinued in 47% of cases with a median treatment duration of 11.5 months, which was shorter compared with previous studies [3,42]. Interestingly, the ORR was similar between the acalabrutinib and idelalisib-R treatment, confirming that toxicity and early drug discontinuation may have contributed to the improved PFS with acalabrutinib. The authors speculated that a higher rate of discontinuation of idelalisib was correlated either to a better clinical experience which had facilitated an earlier identification of AEs, or to the setting of the patients (i.e., younger and less pretreated with a more intact immune system).

Currently, combination of idelalisib and rituximab is indicated in relapsed/refractory (R/R) CLL and in first-line therapy of patients with *del(17p)/TP53* mutations. However, the latter was restricted only to patients not eligible for any other therapies, as alternative treatments have better benefit/risk ratio. Indeed, prolonged follow-up documented serious adverse events during idelalisib administration, including but not limited to a high risk of autoimmune complications (i.e., colitis, pneumonitis and transaminitis) and infections (i.e., *cytomegalovirus* reactivation and *pneumocystis jirovecii* pneumonia) [44]. Current limits to the use of idelalisib in clinical practice together with strategies for overcoming these challenges will be discussed in the following paragraphs.

### 3.2. Duvelisib

Duvelisib (IPI-145) is a dual inhibitor of PI3Kδ and PI3Kγ isoforms (Figure 2) that have been shown to support CLL survival in distinct and independent manners [45]. Preclinical evidence showed that PI3Kδ inhibition directly affects the leukemic B cells, whereas PI3Kγ inhibition targets key support cells in the tumor protective niche, such as CD4^+^ T cells and M2 tumor-associated macrophages [34,46] (Figure 1). The unique binding affinity to PI3Kγ together with a long target residence time represent distinct features compared to idelalisib that may improve the therapeutic profile of duvelisib [47]. Furthermore, duvelisib overcomes the ibrutinib resistance of treatment-induced BTK C481S mutation in vitro [48].

The recommended oral dose of 25 mg BID of duvelisib was identified in the first phase I study as the most appropriate for patient affected by different hematological malignancies, including treatment naïve (TN; n =18) and R/R (n = 55) CLL [49]. Patients remained on treatment for a median of 62.3 and 24 weeks in TN and R/R cohort, respectively. The achievement of a clinical response was higher in TN (83%) than R/R (56%) patients, while being independent from adverse prognostic features in both cohorts. The efficacy of duvelisib monotherapy was confirmed in the phase III “DUO trial” comparing duvelisib to ofatumumab in 319 R/R CLL, excluding previously BCR inhibitors treated patients from trial [50]. After a median follow-up of 22.4 months, duvelisib resulted superior to ofatumumab in terms of PFS and ORR rates (13.3 vs. 9.9 months and 73.8% vs. 45.3%, respectively). The higher benefit of duvelisib was conserved when considering the subset of patients who received ≥2 prior lines of therapy [51], for which the drug obtained the FDA approval in 2018. Similarly to idelalisib, the safety profile of duvelisib lead to a high rate of treatment discontinuation, thus hampering the therapeutic advantage of this molecule.

## 4. Toxicity Affects Current Use of Approved PI3K in CLL

Likewise other B-cell receptor inhibitors, first generation PI3K inhibitors are administered indefinitely until disease progression or intolerable toxicities occur. Patients and doctors need to be aware of potentially life-threatening adverse events that may occur during treatment. Specifically, idelalisib carries a black-box warning for fatal and/or severe colitis, with risk of intestinal perforation, hepatotoxicity, and pneumonitis. Nevertheless, these molecules still have an important role to play in patients who have R/R disease or are intolerant to other CLL target therapies.

### 4.1. Diarrhea and Colitis

Diarrhea is known to be associated with PI3K inhibition, representing one of the most commonly reported AEs with both idelalisib [42,52] and duvelisib [50,51] therapy. Nearly half patients developed any-grade diarrhea, which incidence is higher in TN than in R/R patients. Diarrhea can occur at any time after treatment initiation. An early-onset, less severe diarrhea generally manifests within the first 8 weeks of treatment, while late-onset diarrhea (median time to onset of around 7 months) is mostly grade ≥3 and can be life-threatening [44]. The latter has been reported to be influenced by on-target effects of PI3K p110δ inhibition associated with immune dysregulation of regulatory T cells and CD8 cytotoxic T cells [53]. 

### 4.2. Autoimmune Hepatotoxicity

Multiple lines of evidence point to an autoimmune mechanism causing hepatotoxicity in PI3K-treated CLL. In the case of idelalisib, an elevation of alanine transaminase (ALT) and aspartate transaminase (AST) blood levels of any grade frequently occurs within the first 12 weeks of initiation (35–50%; grade ≥3 in 14–16% of patients) [44,52]. The incidence of hepatotoxicity is higher in patients aged less than 65 years and previously untreated subjects, who are expected to have a more intact immune system [54]. Duvelisib also carries warnings/precautions for hepatotoxicity transaminase elevation that occurs in 8% cases and lead to dose interruption in 6% [50].

### 4.3. Autoimmune Pneumonitis

Noninfectious autoimmune pneumonitis has been reported in 2–5% of patients receiving PI3K inhibitors [3,50]. These patients show increased levels of proinflammatory cytokines (including IFN-γ, IL-6, IL-7, and IL-8) [33] associated with a T-helper 1–type immune response and reported as mediators of pneumonitis. The approval of idelalisb and duvelisib required a black box warning on the potential for pneumonitis-related death.

### 4.4. Rashes and Cutaneous Reactions

Skin adverse drug manifestations under PI3K inhibitors may include exfoliative dermatitis, various rashes, and other skin disorders. Cutaneous reactions have been reported between 3% and 13% of patients receiving PI3K inhibitors, with ≤2.4% reporting severe rash [3,39]. The reported frequency of rash was considerably higher in treatment-naïve patients [42].Current evidence on the mechanism of this complication remains limited.

## 5. Overcoming PI3K Inhibitors Treatment Challenges 

Inhibition of PI3K remains an important strategy in the management of CLL but several issues arising are of similar magnitude as the therapeutic advances that have been made. Targeting the PI3Kδ pathway with idelalisib fell out of favor because of the emergence of severe adverse events and relegated the use of this agent to a salvage therapy after other treatment options have been exhausted. Moreover, despite the remarkable efficacy that was expected with any agents in this class [55], treatment failure was described in few cases with poor outcome [56,57,58]. These obstacles highlight the need for alternative and safer approaches for PI3K inhibition in CLL. The appropriate management of toxicities of current generation PI3K inhibitors will help avoiding treatment discontinuation. Similarly, the use of alternative dosing schedules represents an ongoing effort to improve tolerability and application of PI3Ki therapies in CLL. Moreover, next generation PI3K inhibitors have been recently designed and early data of ongoing clinical trials suggests these agents are highly effective with potentially differentiated toxicity profiles. This would also allow combinations therapy with other novel agents to improve the therapeutic efficacy. 

### 5.1. Correct Management of Toxicity

The key issue with first generation PI3K inhibitors is preventing and managing toxicities over time. Existing guidelines for the use of idelalisib have been established based on expert panel opinion and reviews that are available in multiple publications [44,54]. Information is included in the package insert that comes with the drug. The approaches suggested for idelalisib can reasonably be extrapolated to other PI3K inhibitors as duvelisib. We have summarized recommended management indications for key toxicities below.

All diarrhea developing on PI3K inhibitors require to exclude the presence of causes related to diet, other medications or infections by stool culture testing and clinical monitoring. Drug suspension and antidiarrheal agents are usually successful to manage grade 1–2 early-onset diarrhea. Conversely, the immune-mediated colitis manifests initially as watery, nonbloody diarrhea that responds poorly to antimotility agents, requires treatment interruption and initiation of corticosteroid treatment [44]. Colonoscopy and gastroenterological referrals should be evaluated in the presence of bloody diarrhea and in cases unresponsive to treatment [44,54]. Awareness of the histologic features associated with idelalisib administration is critical to distinguishing idelalisib-associated enterocolitis from potential mimics, including GVHD, autoimmune enterocolitis and infections. In a significant number of cases, idelalisib-associated diarrhea/colitis may be due to an infectious etiology secondary to immune dysfunction [59] suggesting to include in the workup an intestinal biopsy when a pathogen is elusive with standard testing. Median time to resolution is 1 week to 1 month across trials and drug rechallenge is more difficult because of the highly frequent reappearance of this adverse event.

Liver function monitoring is highly recommended every 2 weeks for the first 3 months then every 4 weeks for the next 3 months and every 1 to 3 months thereafter. PI3K therapy should be held in case of ALT and AST levels more than 5 times the upper limit of normal. Transaminitis tends to rapidly remit after drug withdrawal and usually responds to steroids, although it may rapidly recur upon re-exposure suggesting to rstart at a reduced dose after liver function normalization.

The onset of even mild respiratory symptoms under PI3K inhibitor treatment requires extreme caution as they can rapidly evolve to severe deficits in respiratory reserve. PI3K treatment should be stopped in the case of persistent cough, a 5% drop in O_2_ saturation, dyspnea with exertion, or interstitial infiltrates on imaging and empiric prednisone be started while the patient undergoes an infection workup. Evaluation for an infectious etiology, including opportunistic infections such as *pneumocystis jirovecii* pneumonia (PJP) is needed. In case of suspected autoimmune pneumonitis PI3K inhibition should not be restarted.

For cutaneous reactions covering less than 30% of body surface area, with no evidence of superinfection (grade 1–2), the recommendation is to monitor closely. In case of rashes covering more than 30% of body surface or with evidence of superinfection, it is recommended that PI3K inhibition be held, and that dermatologic expert be consulted prior to planning about PI3K inhibition resume. Most patients can be successfully rechallenged. If serious cutaneous reactions reoccur, the drug should be discontinued permanently.

In patients treated with PIK3is, a high rate of serious adverse events (AEs) and increased mortality is mainly associated to infections, in particular PJP and cytomegalovirus (CMV)-related disease. Before starting treatment with PIK3is evaluation of chest X-ray, CMV serological status, HIV, HCV, HBV serological status, pulmonary function tests, and latent TB screening should be performed [54]. As prophylaxis for PJP, trimethoprim-sulfametoxazole is indicated during treatment and up to 6 months after discontinuation in all patients [60]. CMV viremia should be regularly monitored once every two weeks, or at least once a month, and should be performed in any persistent episode of fever. Pre-emptive therapy is recommended for value of CMV-RNA ≥100 000 copies/mL or rising values in two successive controls [54]. Patients with active tuberculosis should be treated before starting therapy with PI3K inhibitors, but treatment should also be performed in patients with latent tuberculosis [61]. Inactivated influenza vaccine and pneumococcal vaccine are strongly recommended. Patients with anti-HBc positivity must receive prophylaxis with lamivudine or entecavir [62]. 

### 5.2. Optimizing Dose Schedules

Novel dosing schedules may represent a valid strategy to optimize tolerability of PI3K inhibitors with potential impact on efficacy. In case of therapy-related adverse events, treatment interruptions are feasible and do not seem to be associated with inferior clinical outcomes, as opposed to BTK inhibitors [63]. Several findings suggest that dose interruptions of 1 week or longer do not significantly impact response to duvelisib in most patients [64]. Additionally, overall survival benefits are maintained in CLL patients experiencing both interruption and dose reduction of idelalisib therapy. In a real-world experience, Bange et al. demonstrated durable treatment responses after toxicity related discontinuation of idelalisib [65]. Most of the treatment-limiting toxicities are immune-mediated and depend on Tregs depletion, mediated by PI3Kδ inhibition [66]. Based on this evidence, it is reasonable to speculate that intermittent dosing schedules with drug free intervals could allow Tregs recovery, thus hopefully improving tolerability of existing PI3Kδ inhibitors. An ongoing phase II trial is currently evaluating such therapeutic strategy and will help to clarify its future application in clinical practice (NCT03961672: phase II study of intermittent dosing of duvelisib in patients with R/R CLL). Immune-related toxicity is typically late in onset, occurring beyond 6 months treatment on average, while disease responses are rapid with a median time of 1.9 months under idelalisib treatment [67]. Thus, a fixed duration therapy limited to less than 6 months may minimize toxicity, thereby allowing combinations with other CLL therapies to improve efficacy. 

### 5.3. Next Generation PI3K Inhibitors

#### 5.3.1. Umbralisib

Umbralisib (TGR-1202) is a next-generation inhibitor of the PI3K isoform p110δ (PI3Kδ) that displays different chemical structure and kinome profiling from that of idelalisib and duvelisib (Figure 2). Dissociation constant determinations indicate an improved PI3Kδ selectivity which is associated with cytochrome P450 inhibition and a high level of plasma exposure allowing a once daily oral administration [68]. The activity of umbralisib is also driven by the additional inhibition of caseinkinase-1ε (CK1ε), a regulator of the WNT signaling and onco-proteins such as c-Myc [68]. This unique capability gives the molecule an additional potential therapeutic efficacy in lymphoma patients characterized by *c-Myc* overexpression, including CLL [68].

The first-in-human phase I study identified the recommended daily (QD) dose of 800 mg, pharmacokinetics and safety of umbralisib in 24 patients with R/R CLL [49]. Preliminary anti-cancer activity was assessed as a secondary endpoint in 20 patients of which 85% achieved objective responses with a median time to best nodal reduction of five cycles. Tumor reductions tended to improve over time. Additionally, umbralisib demonstrated to be clinically active in most of high-risk cytogenetic cases. Mean response duration was 13.4 months associated with a median progression-free survival of 24 months in a post-hoc exploratory analysis of 20 CLL patients. The efficacy of umbralisib monotherapy was confirmed in a phase II study, including 51 CLL patients intolerant to other kinase inhibitors, in which median PFS was not reached after a median follow-up of 9.5 months [69].

Umbralisib treatment was well tolerated across different lymphoid malignancies and demonstrated a differentiated safety profile compared to other PI3Kδ inhibitors. Davids et al. conducted an integrated safety analysis of 347 patients pooled from 5 clinical trials containing umbralisib (42% in monotherapy) administered for lymphoid tumors, including 34% CLL [70]. The most common all-grade toxicities were diarrhea, nausea, and fatigue. The majority of diarrhea events were grade 1 and resolved without any intervention. Hematological adverse events (AEs) included neutropenia (22%), anemia (20%), and thrombocytopenia (18%). Grade 3–4 AEs were uncommon and most frequently represented by neutropenia. Discontinuation of umbralisib due to treatment related adverse events was uncommon, occurring in 7–10% of patients [49,70].

Umbralisib showed possibly fewer immune mediated toxicities than previously observed with other agents of the same class. Specifically, this next generation PI3K inhibitor has been infrequently associated with treatment-related pneumonitis, transaminitis, colitis, which differentiates umbralisib from the toxicity profile of idelalisib [71]. Currently available evidence suggests that this improved safety profile is due to a unique capability to inhibit CK1ε, in addition to modulating the PI3K signaling. Mechanistically, Maharaj et al. demonstrated that umbralisib disables WNT signaling through inhibition of CK1ε and exerts less detrimental effects on Treg immunosuppressive function in CLL [72].

#### 5.3.2. Other Next-Generation PI3K Inhibitors under Clinical Development

A number of next-generation PI3K inhibitor candidates are currently under development. Their pharmaceutical properties are expected to maintain clinical utility while potentially minimizing the immune-related adverse events which have limited the use of first—generation compounds.

The experimental compound zandelisib (ME-401) is an oral, once-daily, selective inhibitor of PI3Kδ allowing the reduction of tumor burden in 89% of R/R CLL/SLL in an ongoing phase Ib trial (NCT02914938). Responses were durable with more than half of patients still responding to treatment after a median follow-up of 7.4 months [73].

YY-20394 is a new type of PI3Kδ selective inhibitor displaying distinct structural characteristic which may decrease the risk of serious infection seen with idelalisib and duvelisib. A phase I, first-in-human, dose escalation study demonstrated that YY-20394 is well tolerated with promised objective response in patients with R/R B-cell malignancies, including a limited number of heavily pretreated CLL (NCT03757000).

In early clinical trials, the selective inhibition of the PI3Kδ isoform by acalisib (GS-9820) and parsaclisib (INCB050465), showed promising activity with a high ORR in CLL patients [74,75]. However, the emergence of late onset toxicity characteristic for PI3Kδ blockade led to some treatment discontinuation, requiring the introduction of a weekly dosing schedule for parsaclib. During this dosing period, tolerability was significantly improved and two out of six CLL patients showed an objective response. 

An additional next-generation PI3Kδ inhibitor called dezapelisib (INCB040093) has been tested alone or in combination with the JAK1 inhibitor itacitinib in a phase I clinical trial in relapsed B-cell lymphomas [76]. Although dezapelisib seems to be highly active in lymphomas, only half of CLL patients responded and all of them with partial response.

Copanlisib (BAY 80-6946) is a pan-class I PI3K inhibitor with preferential activity against the PI3Kα and PI3Kδ isoforms [77]. It demonstrated efficacy in heavily pretreated patients with indolent lymphomas, including a small number of CLL patients in which ORR was 38.5%. The drug is administered intravenously once weekly providing similar pAKT inhibition of continuous orally administered PI3K inhibitors, but with a potential improved tolerability [78] and patient adherence [79,80]. Indeed, copanlisib is associated with less immune-related adverse events than idelalisib and duvelisib, even though other types of AEs such as hyperglycemia and hypertension are common as well as nausea and rash [81]. A trial investigating copanlisib plus nivolumab in patients with Richter transformation (NCT03884998) is currently ongoing.

Voxtalisib (SAR245409, XL765) is a reversible, potent inhibitor of all four classes I PI3Ks and a weaker inhibitor of mTOR. It has been tested in a phase II clinical trial [82] in patients with R/R CLL with a median of four prior anticancer regimens. This agent showed an ORR of 11.4% with a median PFS of 14.4 24.1 weeks. Most frequently reported adverse events were diarrhea (35.3%), fatigue (31.7%) and nausea (26.9%). The most frequently reported grade ≥3 adverse events were anemia (12%), pneumonia (8.4%) and thrombocytopenia (7.8%). 

In a Phase I Trial, pilaralisib (SAR245408/XL147) a novel, highly selective, reversible and potent inhibitor of class I PI3K α, β, γ and δ isoforms demonstrated an acceptable safety profile, consistent with other PI3K inhibitors, and showed preliminary clinical activity [83]. Five of 10 patients with CLL (7 of 10 with high-risk features) had a PR with a PFS ranging from 7.4 to 22.0 months. The most common AEs of any grade were diarrhea (92.0%), pyrexia (52.0%), fatigue (44.0%) and nausea (40.0%). Grade ≥3 AEs were reported in 88.0%, most commonly neutropenia (32.0%), diarrhea (20.0%), anemia (16.0%), and hypotension (12.0%).

## 6. Combinations of PI3K Inhibitors with Other Agents

A number of studies are also evaluating PI3K targeting molecules combined with other novel agents or monoclonal antibodies. The goal of these trials is to evaluate the potential of deeper responses, fixed duration therapy and the chance of prolonged response duration. 

### 6.1. Combination of PI3K Inhibitors and Anti-CD20 Antibody

There exists no unanimous consensus on the combination of PI3Kis and anti-CD20 antibody. Idelalisib has been utilized with rituximab as a heritage of initial studies in R/R CLL that compared the PI3Kis with anti-CD20 antibody. A rational for the use of this combination is that a PI3K inhibitor with rituximab could provide more rapid and deeper remissions (i.e., more patients with undetectable MRD and lower levels of residual cells in bone marrow) than the single agent. Additionally, the effect of PI3Kis on the tumor microenvironment may increase the bone marrow exposure of CLL cells to antibodies, enhancing their efficacy. Therefore, it is possible that in addition to achieving deeper responses more quickly, a long-term PFS benefit might be obtained, even though these findings have indirectly been confirmed only in trials of association of anti-CD20 antibody obinutuzumab and the allied class of BTK inhibitors [84]. Achieving a deeper and more rapid response might provide longer time to progression than other responses if treatment is discontinued, and it could represent a significant advantage for molecules such as PI3Kis in which patients often need to discontinue the treatment before progression. On the other hand, this combination could determine the worsening of a majority of adverse events, in particular infections and cytopenia and the onset of other such as infusion-related reactions, in the contest of a treatment which is already burdened by significant AEs.

Based on the synergistic activity in a preclinical study [85], a doublet combination of the PI3Kdelta inhibitor umbralisib and the novel anti-CD20 monoclonal antibody ublituximab was undertaken in a phase III clinical trial in patients with TN and R/R CLL. Umbralisib plus ublituximab exhibited a well-tolerated safety profile with a treatment discontinuation rate due to AEs of 16.5% and significantly improved PFS compared with obinutuzumab plus chlorambucil standard of care [86].

### 6.2. Combination of PI3K Inhibitors with BTK and BCL-2 Inhibitors 

CLL cells usually have a significant dependence on BCL-2 for survival. In vitro, PI3Kδ inhibition can reverse the protection of stroma on CLL cells which become sensitive to BCL-2 inhibition [87]. Therefore, combinations of PI3K with BCL-2 inhibition might more effectively kill the resistant reservoirs represented by bone marrow (BM)-resident CLL cells, increasing response depth of single agents. In this respect, duvelisib and venetoclax combined treatment resulted in enhanced apoptosis even in CLL cells cultured under conditions that simulate the tumor microenvironment [88]. Based on this finding, a Phase I/II study evaluating this combination of drugs is ongoing (NCT03534323). 

The inhibition of multiple targets in the B-cell receptor (BCR) pathway represents an additional combination strategy to improve the depth and durability of response. Davids et al. investigated dual BCR signaling blockade in a phase I-Ib multicenter trial of umbralisib and ibrutinib association in R/R CLL. The study demonstrated efficacy and tolerability of combined agents associated with the emergence of serious adverse events in 29% of patients [89]. 

Triplet combinations involving umbralisib and ublituximab with either ibrutinib or venetoclax have shown promising activity in CLL. In advanced B-cell malignancies, a phase I study of triplet ublituximab, umbralisib, and ibrutinib demonstrated 84% ORR with manageable grade 3–4 AEs, including neutropenia (22%) and cellulitis (13%) [90]. At the end of 12 cycles of triplet umbralisib, ublituximab, and venetoclax regimen, the objective response rate of R/R CLL was 100%, which included 42% CR by iwCLL criteria. Interestingly, undetectable MRD (<0.01%) in the peripheral blood (PB) and bone marrow (BM) was observed in 95% and 68% of patients respectively [91].

## 7. Conclusions

The intensive basic research and drug development have resulted in significant progresses in PI3K pathway targeting for the treatment of CLL. Inhibition of PI3K remains a relevant therapeutic option in the CLL treatment armamentarium in addition to the more successful use of BTK and BCL-2 antagonists. Indeed, subsets of patients treated with such agents still experience disease relapse with limited therapy alternatives, or result ineligible for their use because of significant comorbidities (e.g., cardiac or renal disease).

To date, a number of PI3K inhibitors demonstrated important anti-CLL activity in clinical trials and general practice. Idelalisib was the forerunner of molecularly targeted agents approved for CLL. However, the emergence of immune-mediated and infectious toxicity led to its reduced use and PI3K inhibition as a treatment paradigm fell out of favor compared to other inhibitors. The prevention and managing of toxicities over time is a key issue with this first generation PI3K inhibitor and, to some extent, with duvelisib that is also associated with mild-to-moderate common side effects. These considerable clinical challenges may be overcome with improved design of novel PI3Kis as umbralisib which is better tolerated than idelalisib. Additionally, the development of novel dosing regimens of both current and next generation PI3K inhibitors may lead to improved tolerability, representing an important therapeutic advance for CLL patients. An alternate strategy may consist in tailoring the duration of PI3Ki administration to prevent the development of late onset toxicities. 

PI3K inhibitor as a monotherapy and in combination with other agents is currently a rapidly evolving field in CLL treatment. These therapeutic approaches may provide new opportunities in patients relapsing after BTK inhibitors or venetoclax [58,92] for which only cellular based regimens represent an option. Chimeric antigen receptor T cell (CAR-T) therapies are currently being studied in patients with high-risk R/R CLL and appear promising [93]. However, many challenges and questions remain unanswered, including the determination of the most effective CAR-T product and how to manage treatment-associated adverse events that could impact the overall feasibility of CAR-T use in elderly CLL patients. Same limits apply to the use of allogeneic stem cell transplantation for which PI3K inhibitors might be useful as bridging therapy.
